# Supplementary material for: Pyruvate Dehydrogenase Kinase 1 inhibition mediated oxidative phosphorylation enhancement in cartilage promotes osteoarthritis progression
Source: BMC Musculoskelet Disord. 2023 Jul 20;24:597. doi: 10.1186/s12891-023-06585-6 (PMC10357736; doi:10.1186/s12891-023-06585-6)
Supplement: Supplementary file 1 — Additional file 1. [file 12891_2023_6585_MOESM1_ESM.zip › MF.Gsea.1653627316242/GOMF_NEUROPEPTIDE_RECEPTOR_ACTIVITY.html]

Details for gene set GOMF\_NEUROPEPTIDE\_RECEPTOR\_ACTIVITY[GSEA]

|  || Dataset | 55457\_collapsed\_to\_symbols.cls.cls #OA\_versus\_normal.cls.cls #OA\_versus\_normal\_repos |
| Phenotype | cls.cls#OA\_versus\_normal\_repos |
| Upregulated in class | normal |
| GeneSet | GOMF\_NEUROPEPTIDE\_RECEPTOR\_ACTIVITY |
| Enrichment Score (ES) | -0.43941823 |
| Normalized Enrichment Score (NES) | -1.4969777 |
| Nominal p-value | 0.03697479 |
| FDR q-value | 0.18279502 |
| FWER p-Value | 1.0 |
Table: GSEA Results Summary

  

Fig 1: Enrichment plot: GOMF\_NEUROPEPTIDE\_RECEPTOR\_ACTIVITY      
 Profile of the Running ES Score & Positions of GeneSet Members on the Rank Ordered List

  

| SYMBOL | TITLE | RANK IN GENE LIST | RANK METRIC SCORE | RUNNING ES | CORE ENRICHMENT || 1 | SSTR4 | somatostatin receptor 4 [Source:HGNC Symbol;Acc:HGNC:11333] | 1597 | 0.414 | -0.0795 | No |
| 2 | GPR143 | G protein-coupled receptor 143 [Source:HGNC Symbol;Acc:HGNC:20145] | 2730 | 0.277 | -0.1376 | No |
| 3 | NPY1R | neuropeptide Y receptor Y1 [Source:HGNC Symbol;Acc:HGNC:7956] | 3249 | 0.226 | -0.1534 | No |
| 4 | TACR3 | tachykinin receptor 3 [Source:HGNC Symbol;Acc:HGNC:11528] | 3527 | 0.200 | -0.1534 | No |
| 5 | SORCS3 | sortilin related VPS10 domain containing receptor 3 [Source:HGNC Symbol;Acc:HGNC:16699] | 3706 | 0.185 | -0.1474 | No |
| 6 | NPY6R | neuropeptide Y receptor Y6 (pseudogene) [Source:HGNC Symbol;Acc:HGNC:7959] | 4728 | 0.093 | -0.2167 | No |
| 7 | GPR37 | G protein-coupled receptor 37 [Source:HGNC Symbol;Acc:HGNC:4494] | 5474 | 0.035 | -0.2708 | No |
| 8 | GAL | galanin and GMAP prepropeptide [Source:HGNC Symbol;Acc:HGNC:4114] | 5599 | 0.024 | -0.2779 | No |
| 9 | NPY5R | neuropeptide Y receptor Y5 [Source:HGNC Symbol;Acc:HGNC:7958] | 6064 | -0.009 | -0.3130 | No |
| 10 | MCHR1 | melanin concentrating hormone receptor 1 [Source:HGNC Symbol;Acc:HGNC:4479] | 6242 | -0.024 | -0.3242 | No |
| 11 | OPRM1 | opioid receptor mu 1 [Source:HGNC Symbol;Acc:HGNC:8156] | 6351 | -0.032 | -0.3292 | No |
| 12 | NTSR2 | neurotensin receptor 2 [Source:HGNC Symbol;Acc:HGNC:8040] | 6557 | -0.046 | -0.3401 | No |
| 13 | MC2R | melanocortin 2 receptor [Source:HGNC Symbol;Acc:HGNC:6930] | 7169 | -0.093 | -0.3776 | No |
| 14 | GRPR | gastrin releasing peptide receptor [Source:HGNC Symbol;Acc:HGNC:4609] | 7361 | -0.107 | -0.3809 | No |
| 15 | BRS3 | bombesin receptor subtype 3 [Source:HGNC Symbol;Acc:HGNC:1113] | 8109 | -0.164 | -0.4213 | Yes |
| 16 | TACR2 | tachykinin receptor 2 [Source:HGNC Symbol;Acc:HGNC:11527] | 8160 | -0.168 | -0.4071 | Yes |
| 17 | NPY2R | neuropeptide Y receptor Y2 [Source:HGNC Symbol;Acc:HGNC:7957] | 8295 | -0.178 | -0.3983 | Yes |
| 18 | HCRTR2 | hypocretin receptor 2 [Source:HGNC Symbol;Acc:HGNC:4849] | 8495 | -0.193 | -0.3929 | Yes |
| 19 | NPFFR1 | neuropeptide FF receptor 1 [Source:HGNC Symbol;Acc:HGNC:17425] | 9058 | -0.236 | -0.4111 | Yes |
| 20 | TACR1 | tachykinin receptor 1 [Source:HGNC Symbol;Acc:HGNC:11526] | 9090 | -0.239 | -0.3878 | Yes |
| 21 | SSTR5 | somatostatin receptor 5 [Source:HGNC Symbol;Acc:HGNC:11334] | 9272 | -0.253 | -0.3745 | Yes |
| 22 | NPBWR2 | neuropeptides B and W receptor 2 [Source:HGNC Symbol;Acc:HGNC:4530] | 9622 | -0.285 | -0.3710 | Yes |
| 23 | GALR3 | galanin receptor 3 [Source:HGNC Symbol;Acc:HGNC:4134] | 9722 | -0.294 | -0.3470 | Yes |
| 24 | SSTR1 | somatostatin receptor 1 [Source:HGNC Symbol;Acc:HGNC:11330] | 9803 | -0.302 | -0.3207 | Yes |
| 25 | NMBR | neuromedin B receptor [Source:HGNC Symbol;Acc:HGNC:7843] | 10503 | -0.373 | -0.3348 | Yes |
| 26 | GALR1 | galanin receptor 1 [Source:HGNC Symbol;Acc:HGNC:4132] | 11851 | -0.560 | -0.3791 | Yes |
| 27 | GALR2 | galanin receptor 2 [Source:HGNC Symbol;Acc:HGNC:4133] | 12072 | -0.612 | -0.3301 | Yes |
| 28 | SSTR2 | somatostatin receptor 2 [Source:HGNC Symbol;Acc:HGNC:11331] | 12181 | -0.639 | -0.2696 | Yes |
| 29 | NTSR1 | neurotensin receptor 1 [Source:HGNC Symbol;Acc:HGNC:8039] | 12677 | -0.849 | -0.2166 | Yes |
| 30 | SSTR3 | somatostatin receptor 3 [Source:HGNC Symbol;Acc:HGNC:11332] | 12779 | -0.936 | -0.1236 | Yes |
| 31 | NMUR1 | neuromedin U receptor 1 [Source:HGNC Symbol;Acc:HGNC:4518] | 12887 | -1.233 | 0.0010 | Yes |
Table: GSEA details [plain text format]

  

Fig 2: GOMF\_NEUROPEPTIDE\_RECEPTOR\_ACTIVITY      
 Blue-Pink O' Gram in the Space of the Analyzed GeneSet

  

Fig 3: GOMF\_NEUROPEPTIDE\_RECEPTOR\_ACTIVITY: Random ES distribution      
 Gene set null distribution of ES for **GOMF\_NEUROPEPTIDE\_RECEPTOR\_ACTIVITY**
